# Learning based motion artifacts processing in fNIRS: a mini review

**DOI:** 10.3389/fnins.2023.1280590

**Published:** 2023-11-08

**Authors:** Yunyi Zhao, Haiming Luo, Jianan Chen, Rui Loureiro, Shufan Yang, Hubin Zhao

**Affiliations:** ^1^HUB of Intelligent Neuro-Engineering, CREATe, IOMS, Division of Surgery and Interventional Science (DSIS), University College London, Stanmore, United Kingdom; ^2^School of Computing, Engineering and Built Environment, Edinburgh Napier University, Edinburgh, United Kingdom

**Keywords:** fNIRS, brain-computer interfaces, motion artifacts, machine learning, deep learning, evaluation matrix

## Abstract

This paper provides a concise review of learning-based motion artifacts (MA) processing methods in functional near-infrared spectroscopy (fNIRS), highlighting the challenges of maintaining optimal contact during subject movement, which can lead to MA and compromise data integrity. Traditional strategies often result in reduced reliability of the hemodynamic response and statistical power. Recognizing the limited number of studies focusing on learning-based MA removal, we examine 315 studies, identifying seven pertinent to our focus area. We discuss the current landscape of learning-based MA correction methods and highlight research gaps. Noting the absence of standard evaluation metrics for quality assessment of MA correction, we suggest a novel framework, integrating signal and model quality considerations and employing metrics like ΔSignal-to-Noise Ratio (ΔSNR), confusion matrix, and Mean Squared Error. This work aims to facilitate the application of learning-based methodologies to fNIRS and improve the accuracy and reliability of neurovascular studies.

## 1. Introduction

Functional near-infrared spectroscopy (fNIRS), a non-invasive neuroimaging technique, leverages near-infrared light measurements scattered through cortical tissues to estimate changes in oxy-hemoglobin (HbO) and deoxy-hemoglobin (HbR) concentrations, yielding insights into neurovascular coupling and cortical activities (Lloyd-Fox et al., 2010; Leff et al., 2011). The technology's relatively high spatial resolution, cost-effectiveness, and portability make it suitable for various applications such as cognitive neuroscience, clinical neurology, personalized healthcare (Rahman et al., 2020), motor rehabilitation (Zhu et al., 2020), cognitive studies (Fishburn et al., 2014; Skau et al., 2019), brain-computer interfaces (Naseer and Hong, 2015), and studies involving subject movements (Vitorio et al., 2017; Nemani et al., 2018, 2019; Pinti et al., 2018; Novi et al., 2020; von Lühmann et al., 2021). The convergence of fNIRS with Brain-Computer Interface (BCI) has paved the way for trans-formative applications, including the restoration of motor functions in individuals with disabilities and the modulation of neuronal activities (Naseer and Hong, 2015). Augmenting fNIRS with other modalities like electroencephalogram (EEG) amplifies its signal diversity, capturing both electrical and hemodynamic activities (Maher et al., 2023). The infusion of deep learning and transfer learning approaches has also augmented the capabilities of fNIRS-based BCIs, underscoring their efficiency and adaptability (Paulmurugan et al., 2021).

Motion artifacts (MA), such as high-frequency spikes (Janani and Sasikala, 2017), slow drifts (Jahani et al., 2018), and baseline intensity shifts (Huang et al., 2022b), can affect fNIRS data during subject movement, compromising the accurate depiction of cortical activity. Current mitigation methods include trial rejection (Cooper et al., 2012) or employing data processing tools like Homer2 (Huppert et al., 2009). Trial rejection can compromise the data reliability and reduce the statistical power of the hemodynamic response (Di Lorenzo et al., 2019). Additionally, data processing tools often require expert intervention and parameters are dependent on specific datasets. To enhance spatial resolution, cortical sensitivity, and robustness, a more general fNIRS MA processing method is essential.

With the rise of learning-based approaches in biomedical engineering, specialized, real-time tools (Wen et al., 2018; Roy et al., 2019; Susan Philip et al., 2023) are increasingly applied to biomedical imaging modalities, including fNIRS. However, studies focusing on learning-based MA removal in fNIRS remain limited. After conducting a review of 315 studies related to fNIRS, Motion Artifact, and Machine Learning. We then pinpointed seven studies that prioritize the processing of MA, aligning with the prevailing research focus on MA corrections. This review aims to highlight the current state of learning-based MA correction, pinpointing research gaps. Notably, a unified evaluation metric for motion correction quality is absent. We propose an evaluation framework that blends signal and model quality, emphasizing datasets with established ground truth.

The structure of the paper unfolds as delineated: Section 2 critically examines diverse methodologies for mitigating motion artifacts in fNIRS, alongside the corresponding evaluative measures. In Section 3, emphasis is placed on championing a standardized General Evaluation Metric, which is introduced through a weighted equation to ensure steadfast evaluations. The conclusive insights are furnished in Section 4, complemented by a recognition of forthcoming endeavors and prospects.

## 2. Current work on learning-based MA removals

**Figure 1:**
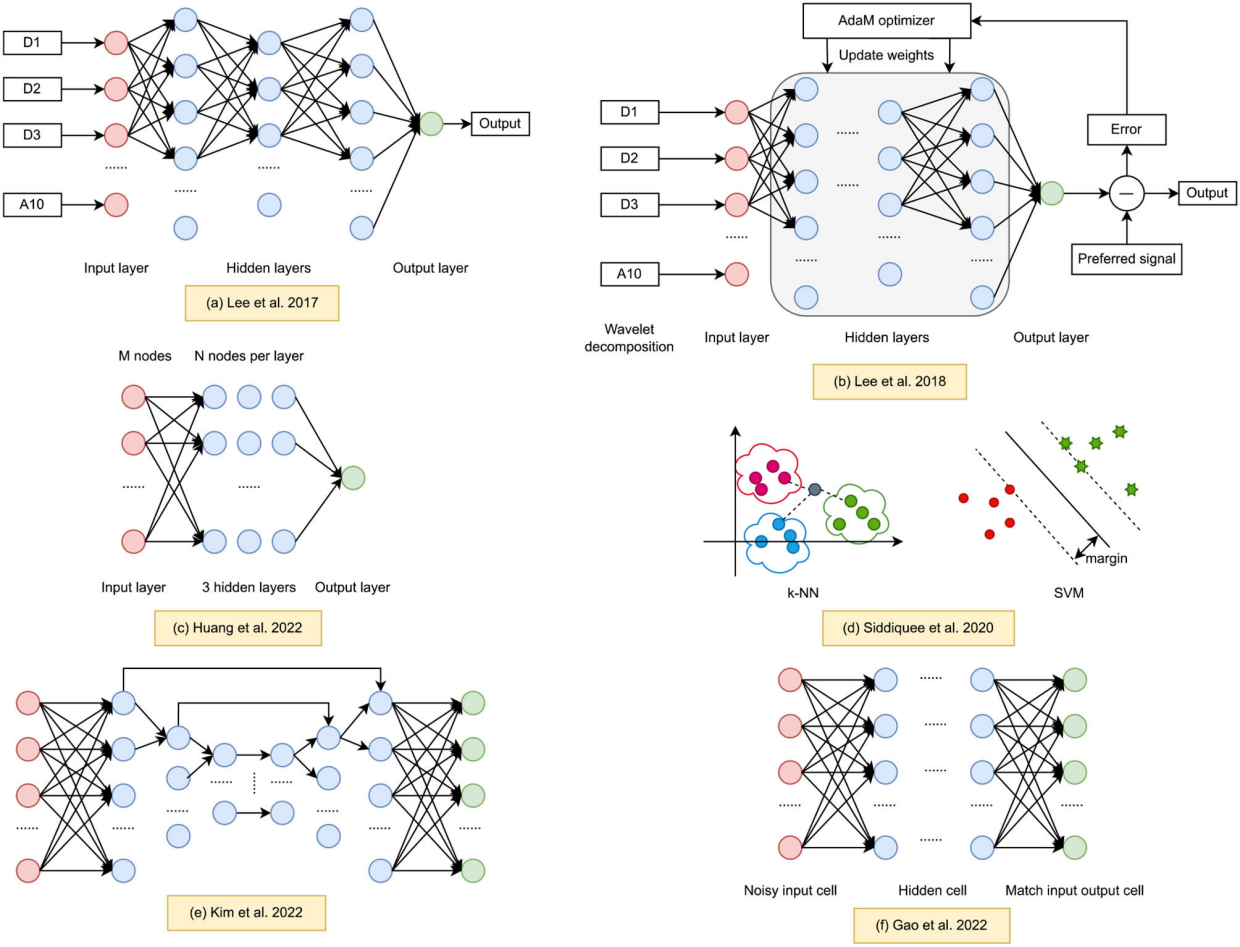
Learning based methods for fNIRS MA processing. **(a)** Wavelet regression ANN (Lee et al., 2017). **(b)** Entropy based cross-correlation BPNN (Lee et al., 2018). **(c)** sResFCNN and low-pass FIR filter (Huang et al., 2022c). **(d)** Machine learning fNIRS MA classifier (Siddiquee et al., 2020). **(e)** U-Net HRF reconstruction (Kim et al., 2022). **(f)** Denoising auto-encoder (Gao et al., 2022).

Deep Neural Networks (DNNs) have recently become prominent tools in biomedical engineering due to their resilience, real-time capabilities, and ability to handle large amounts of data. This section summarizes current research on learning-based MA removal methods, particularly for fNIRS datasets. Our review methodically examined literature based on criteria in Table 1, which details the keyword selection. While many papers concentrated on fNIRS signal processing, we further filtered to focus on MA correction, identifying six relevant papers on learning-based techniques that are particularly useful for large fNIRS datasets. Figure 1 offers an overview of these approaches, illustrating the network architectures proposed in the studies.

**Table 1:** Current works on learning-based MA removal.

**Study**	**Evaluation matrix**	**Dataset**	**Subject type**	**Data type**	**Method**
Lee et al. (2017, 2018)	CNR, ROI	fNIRS data during ambulatory task	Healthy subject	Experimental data	ANN, wavelet regression NN, BPNN
Siddiquee et al. (2020)	Confusion matrix	fNIRS data collected from 19 volunteers	Healthy subject	Experimental data	LDA, SVM, KNN, GBT, Scikit-learn
Kim et al. (2022)	Correlation coefficient (CC), Area under curve (AUC), MSE	fNIRS data collected from 42 subjects	22 male patient, 20 female patient	Experimental data and simulated data	U-Net, CNN
Gao et al. (2022)	MSE, visual inspection	Open-access fNIRS dataset	N/A	Simulated data	DAE, autoregression
Huang et al. (2022c)	SDR, NMSE	Simulated fNIRS dataset	N/A	Simulated data	RNN, sResFCNN

N/A, Not applicable.

### 2.1. Learning-based methods

#### 2.1.1. Artificial neural network

Lee et al. (2017) introduced a MA correction technique using a wavelet regression neural network. The method specifically targets unbalanced optodes, identified by an unbalance index computed from entropy cross-correlation of neighboring channel pairs. Building on this work, the authors proposed a multi-channel fNIRS MA correction method using an ANN for signal reconstruction (Lee et al., 2018). They employed entropy cross-correlation with fNIRS signals to identify contaminated optodes and utilized a back-propagation neural network (BPNN) for MA correction. Experimental validation, including graphical analysis and Contrast-to-Noise Ratio (CNR) from gait tasks, demonstrated the effectiveness of their approach. However, the authors acknowledged limitations in handling extremely poor fNIRS data with prominent artifacts in many channels. The decision algorithm's reliance on optode/channel imbalance hindered its ability to accurately distinguish normal from abnormal fNIRS channels. Consequently, further research is needed to develop a detection algorithm for classifying abnormal fNIRS channels. As the limitations of the ANN were evident, the community moved toward exploring a broader spectrum of algorithms, particularly looking at traditional machine learning classifiers.

#### 2.1.2. Machine learning classifier

Siddiquee et al. (2020) investigated the impact of MA on vigilance level detection during walking compared to seated conditions. Their study aimed to assess if similar results could be obtained in both scenarios. To do this, the authors devised an experimental protocol inducing different vigilance levels while walking and sitting. They employed supervised classification using brain hemodynamic signals to identify vigilance levels and compared the accuracy of vigilance detection to evaluate the effect of MA. Multiple machine learning models, including Linear Discriminant Analysis (LDA), Support Vector Machines (SVM), K-Nearest Neighbors (KNN), and Gradient Boosting Trees (GBT), were employed to find the optimal classifier for binary classification. The comparison revealed that MA significantly reduced the accuracy of vigilance level detection during walking. To address this, the authors implemented a motion sensor-based artifacts estimation and removal method to investigate if removing artifacts could improve vigilance level detection performance. While traditional classifiers showed promise in addressing certain challenges, the rapid progress in deep learning sparked interest in harnessing their capabilities. CNNs emerged as a powerful tool for fNIRS data processing.

#### 2.1.3. Convolutional neural network

Kim et al. (2022) employed a CNN architecture based on the U-net. To create a training and testing data set, variants of the hemodynamic response functions (HRF) were combined with experimental measurements of motion noise to generate a large-scale data set. The neural network was then trained to reconstruct the hemodynamic response linked to neuronal activity while reducing MA. Through a thorough analysis, the authors established that their proposed method yields a more accurate estimate of the task-related HRF than the previously established methods of wavelet decomposition and auto-regressive models. The mean squared error (MSE) and variance of the HRF estimates produced by the CNN were found to be the lowest among all methods considered in the study. These findings were particularly evident when the semi-simulated data contained HRF variants in terms of shape and amplitude. The proposed CNN method enables precise estimation of HRF amplitude and shape while significantly reducing MA and holds great promise for monitoring HRF changes in real-life settings that are subject to excessive MA. As the deep learning domain matured, researchers started to explore other neural network architectures that can further enhance denoising capabilities. Enter the Denoising auto-encoder.

#### 2.1.4. Denoising auto-encoder model

Gao et al. (2022) proposed a deep learning-based MA removal method for fNIRS data. The authors employed a DAE model, equipped with a specialized loss function, for the purpose of eliminating MA. In order to train this deep learning network, they implemented an auto-regression (AR) model to generate a substantial synthetic fNIRS dataset. The efficacy of the DAE methodology was assessed through the utilization of this synthetic dataset and compared against the conventional methods widely used within the fNIRS community. Furthermore, the performance of the DAE was effectively validated through application to open-access experimental datasets (Yücel et al., 2014, 2015). Building upon the successes of DAE and aiming to integrate the benefits of various architectures, researchers turned their attention toward fully connected neural networks, focusing on optimizing network structures.

#### 2.1.5. Fully connected neural network

Huang et al. (2022c) put forward a MA removal approach that is based on a simplified residual fully connected neural network (sResFCNN) and a low-pass finite impulse response filter. Both the sResFCNN and the low-pass finite impulse response (FIR) filter are amenable to online filtering. The optimal structure of the sResFCNN was determined through an analysis of the training loss and testing loss. The proposed filter was compared with MARA, a wavelet-based filter, and the DSMF filter. The results demonstrate that the proposed filter outperforms the wavelet-based filter and is comparable to, or exhibits improved performance in specific scenarios compared to, the dual-stage median filter (DSMF) in terms of noise suppression and signal distortion.

### 2.2. Evaluation metrics

A diverse range of evaluation metrics has been established to assess the performance of artifacts removal solutions, providing insights into noise suppression and signal distortion.

The ΔSignal-to-Noise Ratio (ΔSNR) in MA removal involves analyzing the SNRs at both the input and estimation stages, and utilizing their difference as a performance indicator (Hossain et al., 2022). By evaluating the disparity between the SNR values before and after MA removal, ΔSNR provides valuable insights into the efficacy of the artifacts removal process and its impact on the signal quality.

The CNR quantifies the distinction between the fNIRS signal prior to and during stimulation, reflecting the relative magnitude of the signal change compared to the accompanying noise in a spatial map (Zhou et al., 2020). Thus, the calculated results could be represented visually, providing an intuitive means to identify the occurrence or suppression of MA (Kohno et al., 2007). However, the selection of an appropriate parameter, and the computational resources required for the back-projection to form a spatial map warrant careful consideration.

Artifact power attenuation (APA) refers to the reduction or suppression of unwanted noise or artifacts present in a signal, typically achieved through filtering techniques, with the aim of enhancing the quality of the underlying information (Janani and Sasikala, 2017). The APA method necessitates a meticulous selection of an appropriate high-pass filter to effectively eliminate physiological noise. It is crucial that the chosen filters are consistently applied to both the measured and reference signals.

The “percent root difference” (PRD) evaluation metric determines the percentage difference between the square roots of two signals, serving as a measure of dissimilarity or deviation between them (Dong and Jeong, 2018). In the context of MA removal, the reference signal is commonly selected as the simulated motionless signal.

The within- and between-subject standard deviation method is a statistical approach used to detect potential MA in fNIRS data. It involves calculating within-subject standard deviations for each chromophore to capture variability in single-trial hemodynamic responses due to MA (Di Lorenzo et al., 2019). Comparing two signals entails plotting their standard deviation values on a scatter plot and assessing filtering performance by counting points above or below a specific line.

## 3. Discussion

The enumeration of methods is presented in Table 1, wherein it is observed that different fNIRS datasets were applied. Also, evaluation metrics varied across the studies, with Gao et al. (2022) reporting results through visual inspection and MSE using ground truth, Lee et al. (2017) and Lee et al. (2018) employing CNR and Region of Interest (ROI), Siddiquee et al. (2020) utilizing MA-contaminated signal classification accuracy, Kim et al. (2022) reporting results using CC, AUC, and MSE, and Huang et al. (2022a) adopting Signal Distortion Ratio (SDR) and Normalized MSE (NMSE) for result evaluation. Consequently, due to the diversity of datasets and evaluation metrics, direct comparison of performance across these methods is not feasible. Thus, it is imperative to conduct a systematic comparison to analyze their respective performance.

### 3.1. Model and signal evaluation

The establishment and implementation of a robust General Evaluation Metric (GEM) for Learning-based methods is crucial to systematically gauge the efficacy of learning-based solutions tailored for MA removal. Such a metric would be independent of the particular dataset employed, thereby allowing for an efficient and holistic comparison among various methodologies. The GEM should not merely evaluate the suppression of artifacts within the signal but should also incorporate an assessment of the adapted learning model in terms of robustness, adaptability and accuracy, especially given the rising significance of such models in this domain.

When scrutinizing learning-based models tailored for fNIRS MA rectification, it is indispensable to amalgamate modeling-specific metrics with conventional methodologies. Two paramount tools in this arena are the Confusion Matrix and the MSE. The Confusion Matrix offers a nuanced performance evaluation for MA detection tasks by bifurcating instances into “clean” and “contaminated by MA” based on the model's prognostications. This metric doesn't merely clarify the model's performance but also reveals pertinent insights that could be pivotal for model refinement, including potential enhancements in model architecture or judicious alterations to the dataset to ensure a balanced representation.

In parallel, other metrics, endowed with their distinct attributes and utilities, are instrumental for a thorough evaluation of fNIRS signals. The MSE stands out as a pivotal metric for MA rectification endeavors. It gains prominence when the rectified fNIRS signal is juxtaposed with the reference signal. Particularly, MSE is the preferred evaluation tool for contexts transcending mere categorical classifications, such as when the output is continuous or regression-oriented. By computing the mean squared deviation between the predicted and the reference values, the MSE offers an acute insight into the model's precision and accuracy. In tandem, the Confusion Matrix and the MSE proffer a comprehensive comprehension of the efficiency of learning-based fNIRS MA rectification models, elucidating potential pathways for optimization.

While certain metrics shed light on the overall proficiency of correction techniques, others might encounter impediments in particular scenarios. For instance, representing the CNR's results in a 2D paradigm complicates its numerical integration with other metrics for innovative evaluations. This method is typically adept for inspecting spatial-filtering methodologies, but care must be exercised in the selection of filters. Applying non-uniform filters might inculcate biases, jeopardizing fair comparisons. The PRD demands auxiliary metrics like the Root-mean-square deviation (RMSE) to discern the congruence between signals, and CC to fathom their resemblance. Introducing these metrics augments computational intricacy, which isn't propitious in this context. The within- and between-subject standard deviation methodology, though capable of differentiating neural response variations from MA fluctuations, makes assumptions that may not always hold, rendering it less than ideal as a standalone approach.

Acknowledging the complexity of the evaluation landscape, the ΔSNR metric stands out as especially pertinent for tasks focused on signal regression. In the context of the GEM framework, ΔSNR serves as an instrumental metric for quantitatively assessing the efficacy of artifact removal techniques, thereby facilitating numerically grounded comparisons among various methods. Importantly, ΔSNR is optimally suited for evaluating performance on simulated data, given that these datasets come with ground-truth information, enabling more accurate and reliable assessment. Conversely, for the evaluation of classification models, the ΔSNR metric is not applicable. Instead, the use of a confusion matrix is advocated, as it provides a comprehensive and tailored evaluation criterion explicitly designed for classification tasks. It is crucial to recognize that classification models typically require ground-truth datasets for training and validation, necessitating evaluation methodologies that are synergistic with the intrinsic properties of simulated data.

### 3.2. GEM equation

In the realm of learning-based techniques for mitigating MAs, the evaluation of both signal fidelity and model efficacy stands as a pivotal pursuit. In the work delineated by Yamada et al. (2017), paramount importance is accorded to the SNR as a pivotal metric within the purview of fNIRS-based learning methodologies. Conversely, Gao et al. (2020) accentuate the import of the MSE as a prominent yardstick for evaluating the efficacy of such techniques. Additionally, the salience of the Confusion Matrix in the appraisal of machine learning approaches finds resonance in the exposition by Gabrieli et al. (2021) in the context of fNIRS.

While the MSE metric is frequently utilized to assess the performance of learning-based models, it may also exhibit a correlation with the change in SNR, thereby possibly serving as an ancillary indicator of signal fidelity. The decoupling of these metrics might be considered to mitigate the risk of skewed evaluations of a learning algorithm's effectiveness, especially when both metrics carry positive weightings. For tasks not oriented toward classification, MSE may suffice as the singular evaluation criterion. Considering the scarcity of well-established guidelines regarding the proportional weighting of these evaluation aspects in the realm of classification problems, we suggest the possible introduction of a novel parameter, denoted as “α”. This parameter could be allocated the role of balancing the assessment of signal fidelity, while the remaining weightings may dictate the proportion of focus given to the model's performance evaluation. This nuanced approach might offer a way to harmonize essential facets of signal quality and model performance within the scope of MA removal techniques. Based on these considerations, we tentatively propose a novel evaluation metric, which might be termed the GEM for learning-based methods, and would be mathematically formulated as follows:


(1) $$\text{GEM}=\{\begin{array}{ll} \begin{array}{l} \alpha \times \Delta \text{SNR}+(1-\alpha )\times ConfusionMatrix\\ \text{ is purposed for classification} \end{array} & \text{if the model}\\ \text{MSE} & \text{otherwise} \end{array}$$

where *α* is in range [0, 1]

This tentative evaluation criterion outlines a possibly balanced framework for considering both signal refinement and model effectiveness. However, achieving a universal comparison across various learning-based approaches may remain an elusive goal, considering the plausible variability in weight allocations between the two evaluation dimensions. It is our hope that future research might explore standardized weighting schemes to enable more direct performance comparisons among diverse learning-based methods.

## 4. Conclusion

This review accentuates the burgeoning interest in employing deep learning techniques for mitigating MAs in fNIRS data analyses, attributed to their adaptability and capabilities for real-time processing. These methodologies have shown potential effectiveness across a range of fNIRS datasets, spanning both empirical and simulated data. However, the task of evaluation is rendered complex by the metrics designed for traditional signal processing. This work cautiously suggests an exploratory evaluation approach that amalgamates signal and model metrics through a weighted formula. This may facilitate concurrent assessment of MA suppression and model performance. Despite these advances, the lack of a universally accepted evaluation framework underscores an important avenue for future research. Accordingly, it is imperative that future investigations undertake quantitative evaluation and rigorous statistical analysis to validate the efficacy of this newly proposed metric, thereby fostering a more robust, universally accepted framework for assessment.

## Author contributions

YZ: Writing—original draft, Writing—review & editing, Methodology, Resources. HL: Writing—original draft, Writing—review & editing. JC: Writing—review & editing. RL: Project administration, Writing—review & editing. SY: Writing—review & editing. HZ: Writing—review & editing.
